# Experimental botany in 2017

**DOI:** 10.1093/jxb/erw504

**Published:** 2017-02-15

**Authors:** Christine Raines, Mary Traynor, Jonathan Ingram

**Affiliations:** 1 Editor in Chief, *Journal of Experimental Botany*Department of Biological Sciences, University of Essex, Colchester, CO4 3SQ, UK; 2 Executive Editor, *Journal of Experimental Botany*Bailrigg House, Lancaster University, Lancaster, LA1 4YE, UK; 3 Senior Commissioning Editor/ Science Writer, *Journal of Experimental Botany*Bailrigg House, Lancaster University, Lancaster, LA1 4YE, UK

*Journal of Experimental Botany* (*JXB*) has a long and prestigious history of publishing high-quality papers presenting new findings across a broad range of plant sciences. This year, 2017, marks the journal’s 68th year, and the coming two years leading up to our 70th anniversary will be a particularly exciting period. With a new Editor in Chief five years ago, it was an important point to reflect on scientific direction and the way *JXB* is perceived by our community. A new team of editors was established, and together we not only reviewed the aims and scope of *JXB* but also reaffirmed our commitment to the breadth of plant science underpinned by the experimental approach which is at the heart of the journal. The name, *Journal of Experimental Botany*, embodies this ethos as well now as it did in that first issue in 1950, with papers from such towering figures of 20th century plant biology as Irene Manton, Andrew Benson and Melvin Calvin (Benson and Calvin, 1950; Manton, 1950). Although the name of the journal remains the same, many other changes have occurred, which the team – expert editorial board and staff members – will be reflecting on, and looking forward from, during the coming year. *JXB* has a vital role to play in the ongoing development of plant science research as we approach the 2020s.

## Research themes across plant science

The editorial board has taken a view that *JXB* will have five major research themes. These themes are non-exclusive and dynamic – they will undoubtedly evolve and change as plant science progresses – but you will see that the contents pages of our regular issues are now divided into these areas:

- **Cell biology** – molecular and vesicular trafficking; cell-to-cell communication; the cytoskeleton; cell division; differentiation and death
- **Crop molecular genetics** – trait and gene characterization; molecular analysis; metabolic processes
- **Growth and development** – integration of internal and external cues determining development and architecture; reproductive biology
- **Photosynthesis and metabolism** – photosynthesis; carbon uptake and assimilation; resource allocation; nutrition
- **Plant–environment interactions** – global change; biotic and abiotic stress; symbioses; plant–rhizoflora interactions; mineral nutrition

Flowing from this aim to publish across the plant sciences is our commitment to make the content as accessible as possible to our readership, stimulating the exchange of ideas between disciplines. The eXtra Botany section is of central importance in the mission of the journal to reach out to the plant science community, showcasing not only outstanding research but also the breadth of plant science presented in the articles we publish, which together characterize the journal (Raines, 2016). Over the past year, topics highlighted have ranged across the journal’s research themes, from architecture and morphogenesis, whether at the whole plant level (Perez *et al.*, 2016; Struik, 2016), in the leaf (Routier-Kierzkowska and Kierzkowski, 2016; Sahaf and Sharon, 2016) or in the roots (Kircher and Schopfer, 2016; Scheres and Laskowski, 2016) to the plant cell (Pedrazzini *et al.*, 2016; Strasser, 2016); and from metabolism (Hanson, 2016; Young *et al.*, 2016), biochemistry (Kosma and Rowland, 2016; Schneider *et al.*, 2016) and transport (Sack *et al.*, 2016; Trifiló *et al.*, 2016) to plant defence (Agut *et al.*, 2016; Groen, 2016). Research involving crops continues to be extremely important, from phenotyping in the field (Christopher *et al.*, 2016; Rebetzke *et al.*, 2016) to epigenetics (Giovannoni, 2016; Gouil *et al.*, 2016). This section is also an opportunity to explore wider comment and opinion in our Viewpoint articles (e.g. Blum, 2016; Maron *et al.*, 2016).

*JXB* also further divides annual content into 12 regular and 10 special issues. Special issues give us the opportunity to present articles in a specific topic area, and include both authoritative reviews and original research papers. Editorials which lead these issues are important touchstones – the specialist editors who synthesize and direct the topic area are able to provide a unique perspective. For example, De Coninck and De Smet (2016) in the special issue ‘Plant peptides – taking them to the next level’ provide an overview of the groundbreaking research being conducted into small signalling peptides and their involvement throughout the plant life cycle, as well as an expanding number of peptide–receptor interactions. Similarly, in a very different area, Rebetzke (2016) in ‘From inspiration to impact: delivering value from global root research’ sets out the immense scope of research involving roots over recent years, not only in bringing new understanding of fundamental processes, but in linking these advances to environmental challenges and commercial breeding (see Reyes *et al.*, 2015). Insight articles, furthermore, open up the research in these issues to a wider audience (e.g. Sage, 2016, on Alonso-Cantabrana and von Caemmerer, 2016, looking at the evolutionary rise of C_4_ metabolism in a special issue on photorespiration; or Taleski *et al.*, 2016, on Roberts *et al.*, 2016, examining complex control of lateral roots). We have found that readers highly value these collections as they provide a thorough foundation and cutting-edge update in the area covered. They are also invaluable for teaching purposes. In 2017 we will be making similar online collections of *JXB* content (or virtual issues) available.

## Online platform and digital repository

You may well be reading this editorial from *JXB*’s new-look online home with the updated Oxford Journals platform. This is a significant development from the publisher, Oxford University Press, which will bring a host of benefits for readers. We hope that you find the new presentation unobtrusive and easy to navigate, but it is a work in progress and we will be continuing this development once the main functionality is in place so that it works fully for everyone who uses the journal. If you have any comments, these are very welcome – just email the editorial office at j.exp.bot@lancaster.ac.uk with any feedback and/or questions.

New services for *JXB* authors that have recently been introduced include full integration of our submission system with Dryad Digital Repository, and this will soon also include the BioRxiv preprint server. *JXB* encourages all authors to deposit the data underlying the results and conclusions presented in a paper, as well as supplementary data, in any publicly accessible archive that guarantees access and preservation. This is another important step forward, and in order to support and encourage such practice we provide full financial support for up to 20 GB data storage in Dryad. In response to calls from authors, we now also accept papers in any reasonable format for initial review and it will shortly be possible to submit papers directly from BioRxiv at the click of a mouse.

## A community journal

We would like to take this opportunity to remind all potential authors that *JXB* does not levy page charges on authors. Our unique open access publication policy is also free to anyone publishing original research in the journal who is a member of a subscribing library or institution. Colour print is also free if you become a member of the Society for Experimental Biology (SEB) – a great deal, and just one of the many benefits of the journal’s close connection with the society and dedication over many years to providing a responsible publishing outlet.

We very much hope that you find *JXB* interesting and enjoyable reading in 2017.
